# Predictive Value of the Log Odds of Negative Lymph Nodes/T Stage as a Novel Prognostic Factor in Bladder Cancer Patients After Radical Cystectomy

**DOI:** 10.3389/fonc.2022.895413

**Published:** 2022-07-19

**Authors:** Tao Chen, Xiangpeng Zhan, Xinpeng Chen, Ming Jiang, Hao Wan, Bin Fu, Luyao Chen

**Affiliations:** ^1^ Department of Urology, the First Affiliated Hospital of Nanchang University, Nanchang City, China; ^2^ The First Clinical Medical College of Nanchang University, Nanchang City, China; ^3^ Jiangxi Institute of Urology, Nanchang City, China

**Keywords:** bladder cancer, nomogram, log odds of negative lymph nodes/T stage, prognosis, SEER

## Abstract

**Background:**

The effect of lymph node resection on the prognosis of bladder cancer (BLCA) patients receiving radical cystectomy should not be ignored. Our aim was to explore the prognostic value of the log odds of negative lymph nodes/T stage (LONT) and construct a more effective nomogram based on LONT to predict cancer-specific survival (CSS) in postoperative BLCA patients.

**Methods:**

Patients diagnosed with BLCA after radical cystectomy between 2004 and 2015 in the Surveillance, Epidemiology, and End Results (SEER) database were enrolled. We randomly split (7:3) these patients into the primary cohort and internal validation cohort. 86 patients from the First Affiliated Hospital of Nanchang University were collected as the external validation set. Univariate and multivariate cox regression analyses were carried out to seek prognostic factors of postoperative BLCA patients. According to these significantly prognostic factors, a simple-to-use nomogram was established for predicting CSS. Their performances were evaluated by using calibration curves, the concordance index (C-index), the receiver operating characteristic (ROC) curves, and decision curve analysis (DCA). In addition, different risk groups were tested by Kaplan-Meier curves and log-rank tests.

**Result:**

Whether in cancer-specific survival (CSS) or overall survival (OS), LONT was an independent and significant prognostic factor. Through further screening, the ultimate nomogram of CSS was composed of nine independent prognostic factors including LONT, age, race, tumor size, histologic type, T stage, N stage, summary stage and chemotherapy. The C-index of nomogram in the primary cohort, internal and external validation cohort were 0.734, 0.720 and 0.728, respectively. The AUC of predicting CSS at 3 and 5 years were 0.783 and 0.774 in the primary cohort and 0.781 and 0.781 in the validation cohort. The results of calibration and DCA showed good concordance and clinical applicability. Significant differences (P < 0.05) were displayed in CSS among different risk groups.

**Conclusion:**

LONT was regarded as a novel and reliable prognostic factor. Compared with the AJCC staging system, the established nomogram based on LONT can more effectively predict the prognosis of BLCA patients after radical cystectomy.

## Introduction

Bladder cancer (BLCA) is the second most common urinary tract malignancy worldwide, whose incidence rate is increasing year by year. According to the global cancer statistics, an estimated 573,278 new BLCA cases and almost 212,536 BLCA deaths occurred in 2020 (1). For muscle-invasive nonmetastatic (MIBC) and high-risk non-muscle-invasive BLCA, radical cystectomy (RC) and pelvic lymph node dissection are often recommended (2). Accurate survival prediction after RC is essential, which is instrumental in guiding postoperative therapy and follow-up. As the most common staging system for evaluating tumor prognosis, the eighth edition of the American Joint Committee on Cancer (AJCC) staging system is based on the primary tumor (T) status, regional lymph node (N), and distant metastasis (TNM) stage (3). However, there is a certain degree of inadequacy, such as neglect of the influence of lymph node dissection (LND), which can lead to the different prognosis of patients with the same TNM.

In recent years, many studies have been devoted to exploring the impact of lymph node dissection on tumor prognosis (4–8). A meta-analysis showed that extended pelvic lymph node dissection (ePLND) brought recurrence-free survival (RFS) benefits compared with non-ePLND. Not only did patients with lymph node positive and pT3-4 diseases benefit from ePLND, ePLND also provided better RFS for patients with lymph node negative (9). Wright et al. thought that the increased number of lymph nodes removed (ELNs) during cystectomy was related to the improvement of the survival rate of patients with positive-lymph node cancer (10). Similarly, Wang et al. found that the number of LND may be an independent factor related to the survival of patients receiving RC (11). In addition, Jin et al. believed that LODDS defined as the log ratio between the number of metastatic lymph nodes and the number of negative lymph nodes had a significant prognostic value for MIBC patients (12). However, the lack of information on individualized tumor characteristics were their common limitation.

In fact, the T stage is a powerful risk factor for BLCA and represents the major tumor features. An increasing number of studies have suggested that T stage is closely associated with prognosis and tumor biological features (13–15). T stage can represent the biological characteristics of tumor. Xie et al. used the combination of negative lymph nodes (NLNs) and T stage (log (NLNs+1)/T stage) as a new prognostic factor to reflect the degree of individualized LND in gastric cancer patients (16). However, whether this novel indicator can be applied to bladder cancer remains unknown.

Here, in this study, we obtained the BLCA receiving RC cohort from the Surveillance, Epidemiology and End Results (SEER) database to explore the prognostic value of this new factor (log (NLNs+1)/T stage) in BLCA patients. And Based on this factor, a more accurate prognostic model for BLCA after radical cystectomy was constructed.

## Materials and Methods

### Population Selection

The SEER database was utilized to obtain all patient records, which is an open-access database covering about 35% of the U.S. population. All patients were enrolled if they met the following criteria: (a) BLCA patients diagnosed from 2004 to 2015. (b) histological subtype: Transitional cell carcinoma. (c) receiving radical cystectomy and lymph node dissection patients. (d) BLCA was the only first primary malignancy. The following exclusion criteria were used: (a) number of lymph nodes removed and positive unknown. (b) T stage and Grade unknown. (c) race and marital status unknown. (d) unclear tumor size. (e) cause of death and follow-up time unknown. The detailed screening process was displayed in **Supplementary Figure 1**.

The external validation cohort was composed of 86 BLCA patients receiving radical cystectomy and lymph node dissection at the First Affiliated Hospital of Nanchang University between 2010 and 2020. The inclusion and exclusion criteria are in accordance with the SEER database. The last follow-up time was February 2022. And our study was approved by the Ethics Committee of the First Affiliated Hospital of Nanchang University.

### Variables Collection

The information of variables collected in this study included the following aspects: demographic characteristics (age, sex, race and marital status), cancer characteristics (T stage, N stage, AJCC stage, Grade, histological type and tumor size), treatment characteristics (radiotherapy and chemotherapy) and other characteristics (regional nodes positive and regional nodes examined). For continuous variables such as age and tumor size, we utilized X-tile software to obtain the best cutoff value (17). The optimal age cutoff values were 63 and 76 years old, which were divided into three groups: <63 years, 63-76 years and >76 years. The optimal tumor size cutoff values were 30 and 64 mm, which were divided into three groups: <30mm, 30-64mm and >64mm (**Supplementary Figure 2**). cancer-specific mortality (CSM) represented the main endpoint, which referred to the death due to bladder cancer.

### Statistical Analysis

In our study, T1, T2, T3, T4 were designated as 1, 2, 3, 4, respectively. LONT was regarded as a continuous variable and defined as log (NLNs + 1)/T stage, where NLNs is the count of regional nodes examined minus the count of regional nodes positive. In order to avoid the phenomenon of zero, we added 1 to NLNs (18). Categorical variables were expressed as totals and percentages, while continuous variables were expressed as medians and interquartile range (IQR). Univariate and multivariate Cox regression models were utilized to determine variables independently related to CSS and OS. Based on these significant variables, we constructed the final nomogram. The performance of the model was evaluated by C-index, the receiver operating characteristic (ROC) curves with the calculated area under the curve (AUC), calibration curve and decision curve analysis (DCA). According to the score of the nomogram, we calculated the cut-off value through X-tile and divided the patients into high-risk, medium-risk and low-risk groups. The difference between the three groups was evaluated by Kaplan-Meier (K-M) curves and log-rank test. All statistical analyses were carried out by R software (version 4.1.0) and SPSS 22.0 (IBM). We considered P-value <0.05 (two-sided) as statistical significance.

## Results

### Patient Characteristics

We enrolled a total of 4610 BLCA receiving RC patients from the SEER database, which included 3227 patients in the primary cohort and 1383 patients in the internal validation cohort. The baseline characteristics of the included population were exhibited in **Table 1**. There was no statistical difference in the clinical variables between the primary cohort and internal validation cohort. Among all BLCA patients receiving RC, the median ELN, NLN and LONT count (IQR) were 14 (8-24), 13 (7-23) and 0.7 (0.5-1.0), respectively. The majority of them were between 63 and 76 years old (44.6%), male (66.7%), white (86.8%) and married (61.1%). Moreover, the most common histologic type and Grade were transitional cell carcinoma (66.1%) and Grade IV (67.7%). Most of the BLCA patients presented with T2/3 (76%) and N0 (69.5%). In terms of treatment, there were 2092 (45.4%) patients who received chemotherapy and 130 (2.8%) patients who received radiotherapy.

**Table 1:** Baseline clinical characteristics of patients.

Variables	All patients (%) 4610	Primary cohort (%) 3227	Validation cohort (%) 1383	p
Median NLN count (IQR)	13 (7-23)	13 (7-22)	14 (7-24)	0.251
Median LONT (IQR)	0.7 (0.5-1.0)	0.7 (0.5-1.0)	0.8 (0.5-1.0)	0.419
Age				0.621
<63	1729 (37.5)	1225 (38.0)	504 (36.4)	
63-76	2054 (44.6)	1427 (44.2)	627 (45.3)	
>76	827 (17.9)	575 (17.8)	252 (18.2)	
Sex				0.170
Male	3077 (66.7)	2174 (67.4)	903 (65.3)	
Female	1533 (33.3)	1053 (32.6)	480 (34.7)	
Race				0.146
White	4002 (86.8)	2820 (87.4)	1182 (85.5)	
Black	306 (6.6)	200 (6.2)	106 (7.7)	
Other^+^	302 (6.6)	207 (6.4)	95 (6.9)	
Marital status				0.544
Married	2818 (61.1)	1965 (60.9)	853 (61.7)	
Unmarried	619 (13.4)	445 (13.8)	174 (12.6)	
SDW	1173 (25.4)	817 (25.3)	356 (25.7)	
Histologic type				0.781
TCC	3047 (66.1)	2137 (66.2)	910 (65.8)	
PTCC	1563 (33.9)	1090 (33.8)	473 (34.2)	
Grade				0.229
I	20 (0.4)	16 (0.5)	4 (0.3)	
II	124 (2.7)	87 (2.7)	37 (2.7)	
III	1347 (29.2)	968 (30.0)	379 (27.4)	
IV	3119 (67.7)	2156 (66.8)	963 (69.6)	
T stage				0.810
T1	365 (7.9)	250 (7.7)	115 (8.3)	
T2	1699 (36.9)	1193 (37.0)	506 (36.6)	
T3	1803 (39.1)	1271 (39.4)	532 (38.5)	
T4	743 (16.1)	513 (15.9)	230 (16.6)	
N stage				0.729
N0	3202 (69.5)	2251 (69.8)	951 (68.8)	
N1	698 (15.1)	480 (14.9)	218 (15.8)	
N2	695 (15.1)	486 (15.0)	209 (14.9)	
N3	15 (0.3)	10 (0.3)	5 (0.4)	
AJCC stage				0.840
I	340 (7.4)	236 (7.3)	104 (7.5)	
II	1425 (30.9)	1004 (31.1)	421 (30.4)	
III	1401 (30.4)	988 (30.6)	413 (29.9)	
IV	1444 (31.3)	999 (31.0)	445 (32.3)	
Summary stage				0.885
Localized	1730 (37.5)	1214 (37.6)	516 (37.3)	
Regional	2794 (60.6)	1951 (60.5)	843 (61.0)	
Distant	86 (1.9)	62 (1.9)	24 (1.7)	
Tumor size				0.893
<30mm	1225 (26.6)	855 (26.5)	370 (26.8)	
30-64mm	2500 (54.2)	1757 (54.4)	743 (53.7)	
>64mm	885 (19.2)	615 (19.1)	270 (19.5)	
Chemotherapy				0.423
No	2518 (54.6)	1775 (55.0)	743 (53.7)	
Yes	2092 (45.4)	1452 (45.0)	640 (46.3)	
Radiotherapy				0.846
No	4480 (97.2)	3135 (97.1)	1345 (97.3)	
Yes	130 (2.8)	92 (2.9)	38 (2.7)	

NLNs, negative lymph nodes; ELNs, Examined lymph nodes; LONT, Log odds of negative lymph nodes/T stage; IQR, interquartile range; Other^+^ American/Indian/Alaska/Native/Asian/Pacific Islands; SDW separated, divorced or widowed; TCC, Transitional cell carcinoma; PTCC, papillary Transitional cell carcinoma.

In the external validation cohort from our medical center, the median ELN, NLN and LONT count (IQR) were 17 (12-25), 16 (11-24) and 0.8 (0.6-1.0), respectively. In addition, the majority of them were between 63 and 76 years old (59.3%), male (81.4%), and married (62.8%). Most of them presented with Grade III/IV (94.2%), T2/3 (81.4%) and N0 (62.8%). Regarding the treatment, the vast majority of people did not receive radiotherapy (97.7%), and about a quarter received chemotherapy (24.4%). Clinical data of patients from our medical center are listed in **Table 2**.

**Table 2:** Demographics and clinical characteristics of the 86 BLCA patients from our medical center.

Variables	Our medical center
	External validation cohort
Median ELN count (IQR)	17 (12-25)
Median NLN count (IQR)	16 (11-24)
Median LONT count (IQR)	0.8 (0.6-1.0)
Age	
<63	21 (24.4)
63-76	51 (59.3)
>76	14 (16.3)
Sex	
Male	70 (81.4)
Female	16 (18.6)
Marital status	
Married	54 (62.8)
Unmarried	6 (7.0)
SDW	26 (30.2)
Grade	
I/II	5 (5.8)
III/IV	81 (94.2)
T stage	
T2	35 (40.7)
T3	35 (40.7)
T4	16 (18.6)
N stage	
N0	54 (62.8)
N1	13 (15.1)
N2	19 (22.1)
AJCC stage	
II	28 (32.6)
III	26 (30.2)
IV	32 (37.2)
Summary stage	
Localized	29 (33.7)
Regional	57 (66.3)
Tumor size	
<30mm	20 (23.3)
30-64mm	49 (57.0)
>64mm	17 (19.8)
Chemotherapy	
No	65 (75.6)
Yes	21 (24.4)
Radiotherapy	
No	84 (97.7)
Yes	2 (2.3)

NLNs, negative lymph nodes; ELNs, Examined lymph nodes; LONT, Log odds of negative lymph nodes/T stage; IQR, interquartile range; SDW, separated, divorced or widowed.

### Effect of LONT on the Prognosis of OS and CSS in Primary Cohort

**Figure 1:**
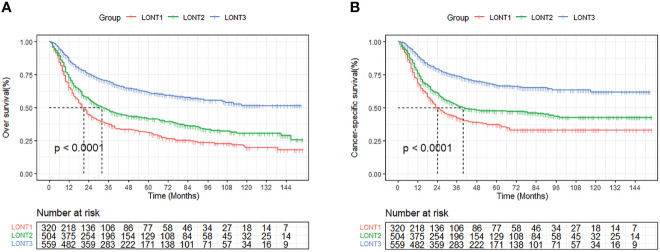
Kaplan-Meier curves of OS **(A)** and CSS **(B)** stratified by the LONT stage.

In order to determine the prognostic factors of BLCA patients after RC, univariate and multivariate COX regression models were carried out. Our results showed that LONT was statistically significant in both univariate and multivariate Cox regression analysis. In addition to LONT, age, race, histologic type, T stage, N stage, summary stage, tumor size and chemotherapy were independent prognostic factors in CSS (**Table 3**). Similar results were also found in OS cohort (**Table 4**). Then we used X-tile software to calculate the cut-off value of LONT and divide it into three groups: LONT1 (-0.6≤LONT ≤ 0.4), LONT2 (0.4<LONT<0.9) and LONT3 (0.9≤LONT ≤ 2.0) (**Supplementary Figure 3**). Kaplan-Meier survival analysis and log-rank test displayed that the prognosis of BLCA patients after RC could be significantly stratified by LONT whether in CSS or OS cohorts (p < 0.001, **Figure 1**). All these results showed that the lower the LONT value, the worse the prognosis of patients.

**Table 3:** Univariate and multivariate Cox regression analyses for overall survival in the primary cohort.

Variables	Univariate analysis			Multivariate analysis		
	OR	95%CI	P	OR	95%CI	P
NLNs^a^	0.983	0.979-0.987	**<0.001**	–	–	–
LONT^a^	0.354	0.317-0.396	**<0.001**	0.626	0.552-0.710	**<0.001**
Age						
<63	Reference			Reference		
63-76	1.238	1.107-1.384	**<0.001**	1.295	1.155-1.453	**<0.001**
>76	2.000	1.754-2.279	**<0.001**	1.822	1.584-2.096	**<0.001**
Sex						
Male	Reference			Reference		
Female	1.175	1.061-1.300	**0.002**	1.020	0.917-1.135	0.721
Race						
White	Reference			Reference		
Black	1.479	1.228-1.780	**<0.001**	1.334	1.101-1.616	**0.003**
Other^+^	0.873	0.727-1.097	0.282	0.907	0.737-1.115	0.354
Marital status						
Married	Reference			Reference		
Unmarried	1.113	0.963-1.286	0.147	1.215	1.045-1.413	**0.011**
SDW	1.224	1.095-1.369	**<0.001**	1.070	0.951-1.204	0.260
Histologic type						
TCC	Reference			Reference		
PTCC	0.702	0.631-0.781	**<0.001**	0.858	0.769-0.958	**0.006**
Grade						
I	Reference			–		
II	1.975	0.848-4.602	0.115	–	–	–
III	2.068	0.925-4.623	0.077	–	–	–
IV	1.780	0.798-3.972	0.159	–	–	–
T stage						
T1	Reference			Reference		
T2	1.426	1.098-1.853	**0.008**	1.139	0.870-1.492	0.343
T3	3.370	2.616-4.340	**<0.001**	1.471	1.089-2.043	**0.021**
T4	5.202	3.997-6.770	**<0.001**	1.860	1.321-2.617	**<0.001**
N stage						
N0	Reference			Reference		
N1	2.171	1.913-2.463	**<0.001**	1.705	1.478-1.968	**<0.001**
N2/3	3.167	2.810-3.569	**<0.001**	2.327	2.024-2.675	**<0.001**
Summary stage						
Localized	Reference			Reference		
Regional	3.201	2.841-3.607	**<0.001**	1.478	1.171-1.865	**<0.001**
Distant	6.805	5.079-9.117	**<0.001**	2.407	1.647-3.518	**<0.001**
Tumor size						
<30mm	Reference			Reference		
30-64mm	1.278	1.134-1.440	**<0.001**	1.096	0.971-1.238	0.137
>64mm	1.695	1.468-1.957	**<0.001**	1.352	1.165-1.570	**<0.001**
Chemotherapy						
No	Reference			Reference		
Yes	0.924	0.838-1.019	0.113	0.711	0.638-0.792	**<0.001**
Radiotherapy						
No	Reference			Reference		
Yes	1.544	1.190-2.002	**0.001**	0.943	0.722-1.232	0.667

CI, Confidence interval; OR, Odds ratio; NLNs, negative lymph nodes; LONT, Log odds of negative lymph nodes/T stage; other^†^American/Indian/Alaska/Native/Asian/Pacific Islands; SDW, separated, divorced or widowed; TCC, Transitional cell carcinoma; PTCC, papillary Transitional cell carcinoma. ^a^These variables were treated as continuous data.

**Table 4:** Univariate and multivariate Cox regression analyses for cancer-specific survival in the primary cohort.

Variables	Univariate analysis			Multivariate analysis		
	OR	95%CI	P	OR	95%CI	P
NLNs^a^	0.984	0.979-0.988	**<0.001**	–	–	–
LONT^a^	0.339	0.299-0.384	**<0.001**	0.633	0.553-0.730	**<0.001**
Age						
<63	Reference			Reference		
63-76	1.152	1.020-1.301	**0.023**	1.224	1.080-1.390	**0.001**
>76	1.597	1.374-1.855	**<0.001**	1.485	1.265-1.743	**<0.001**
Sex						
Male	Reference			Reference		
Female	1.211	1.082-1.356	**<0.001**	1.075	0.955-1.211	0.236
Race						
White	Reference			Reference		
Black	1.628	1.334-1.987	**<0.001**	1.417	1.153-1.743	**<0.001**
Other	0.875	0.692-1.105	0.261	0.891	0.704-1.127	0.336
Marital status						
Married	Reference			Reference		
Unmarried	1.090	0.927-1.281	0.296	1.135	0.959-1.343	0.141
SDW	1.140	1.005-1.294	**0.042**	0.993	0.870-1.135	0.920
Histologic type						
TCC	Reference			Reference		
PTCC	0.675	0.596-0.761	**<0.001**	0.858	0.759-0.971	0.015
Grade						
I	Reference			–		
II	2.956	0.913-9.578	0.071	–	–	–
III	3.419	1.099-10.641	0.051	–	–	–
IV	2.799	0.901-8.696	0.075	–	–	–
T stage						
T1	Reference			Reference		
T2	1.730	1.239-2.416	**0.001**	1.350	0.959-1.900	0.085
T3	4.556	3.298-6.295	**<0.001**	1.809	1.217-2.690	**0.003**
T4	7.199	5.162-10.040	<0.001^*^	2.257	1.498-3.400	**<0.001**
N stage						
N0	Reference			Reference		
N1	2.513	2.187-2.887	**<0.001**	1.854	1.585-2.168	**<0.001**
N2/3	3.715	3.261-4.231	**<0.001**	2.560	2.198-2.980	**<0.001**
Summary stage						
Localized	Reference			Reference		
Regional	3.881	3.365-4.475	**<0.001**	1.611	1.240-2.092	**<0.001**
Distant	9.470	6.951-12.902	**<0.001**	2.834	1.887-4.258	<0.001^*^
Tumor size						
<30mm	Reference			Reference		
30-64mm	1.359	1.186-1.556	**<0.001**	1.148	0.989-1.318	**0.050**
>64mm	1.843	1.568-2.166	**<0.001**	1.456	1.231-1.722	**<0.001**
Chemotherapy						
No	Reference			Reference		
Yes	1.041	0.934-1.160	0.471	0.734	0.651-0.827	**<0.001**
Radiotherapy						
No	Reference			Reference		
Yes	1.660	1.255-2.197	**<0.001**	0.946	0.710-1.261	0.705

CI Confidence interval; OR Odds ratio; NLNs negative lymph nodes; LONT Log odds of negative lymph nodes/T stage; other^†^American/Indian/Alaska/Native/Asian/Pacific Islands; SDW separated, divorced or widowed; TCC, Transitional cell carcinoma; PTCC, papillary Transitional cell carcinoma.^a^ These variables were treated as continuous data.

### Development and Validation of Nomogram

**Figure 2:**
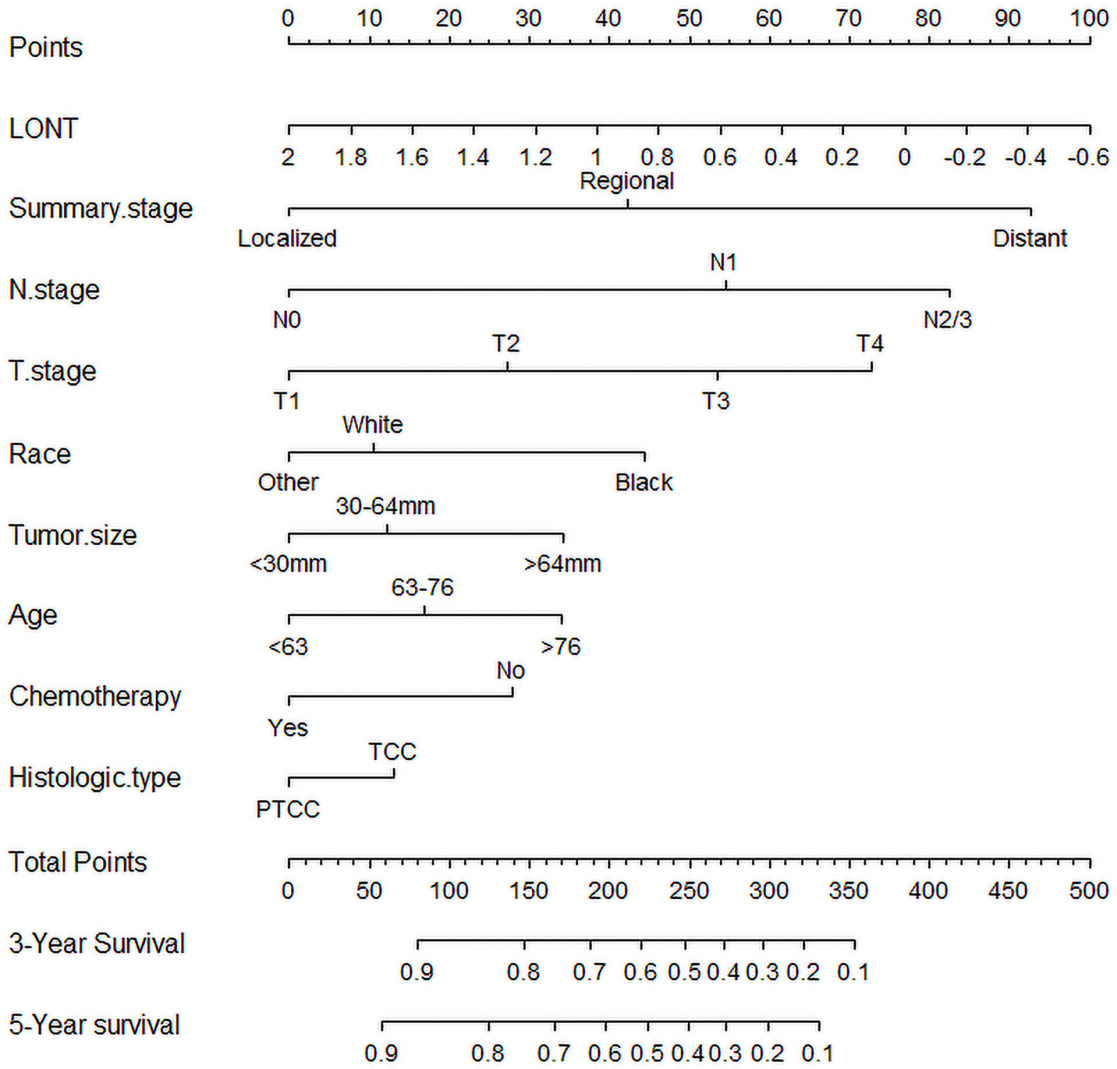
Nomogram for predicting 3-, and 5-year cancer-specific survival rate of bladder cancer patients after radical cystectomy.

**Figure 3:**
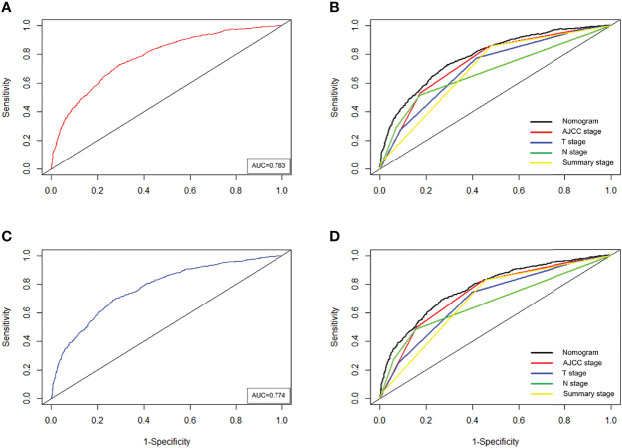
Time-dependent ROC curves of the nomogram predicting 3-years **(A)**, and 5-years **(C)** CSS in the primary cohort. Comparison of the ROC curves between nomogram and other independent factors at the 3-years **(B)**, and 5-years **(D)** CSS in the primary cohort.

**Figure 4:**
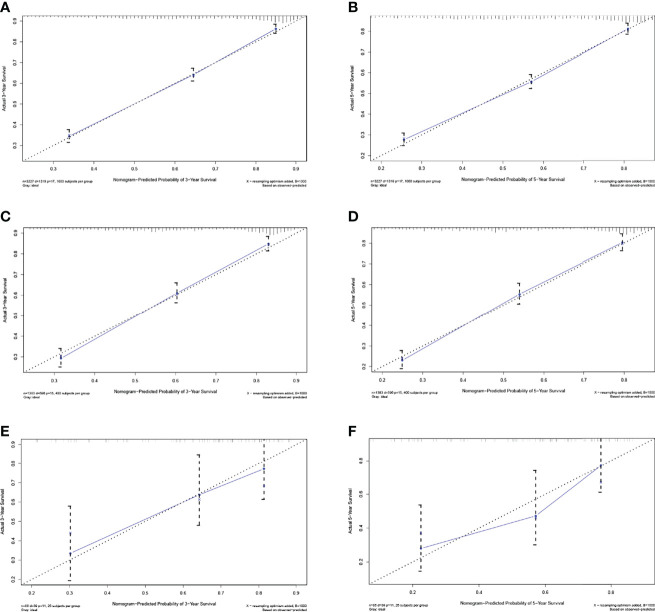
The calibration curves of CSS nomogram at 3- **(A)**, and 5-years **(B)** in the primary cohort, at 3- **(C)**, and 5-years **(D)** in the internal validation cohort and at 3 **(E)**, and 5-year **(F)** in the external validation cohort.

**Figure 5:**
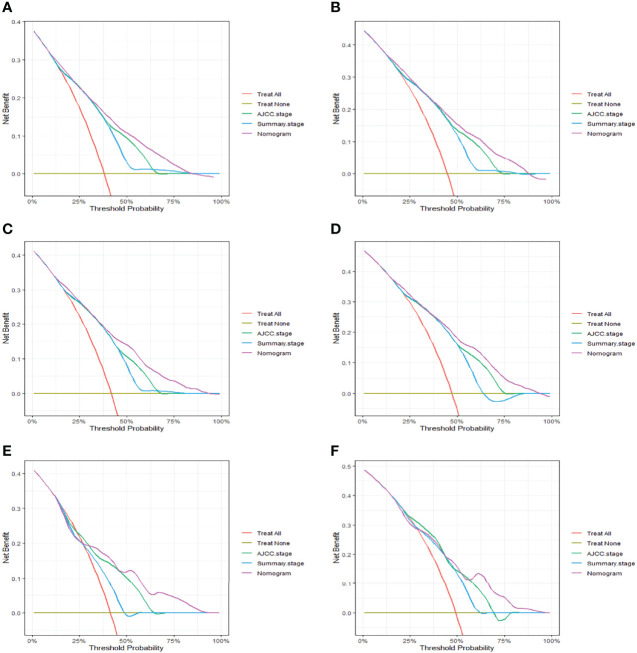
The decision curve analysis (DCA) of CSS nomogram at 3- **(A)**, and 5-years **(B)** in the primary cohort, at 3- **(C)**, and 5-years **(D)** in the internal validation cohort and at 3 **(E)**, and 5-year **(F)** in the external validation cohort.

Based on the above 8 independent risk factors, we constructed a simple-to use nomogram to predict BLCA patients’ CSS. As shown in the nomogram, LONT had the greatest impact on patients’ survival outcome, followed by summary stage, N stage and T stage (**Figure 2**). We can add the scores of each variable in the nomogram to obtain the total score, so as to calculate the survival probability. In addition, we further evaluated the performance of nomogram through C-index, AUC, calibration curve and DCA. The C-index of the primary group, the internal and external validation group were 0.734 (95% CI 0.720 – 0.748), 0.720 (95% CI 0.698 – 0.741) and 0.728 (95% CI 0.655-0.800), respectively. The 3‐, and 5‐year AUC values were 0.783 and 0.774 in the primary cohort (**Figures 3A**, **C**), 0.781 and 0.781 in the internal validation cohort (**Supplementary Figures 4A**, **C**) and 0.722 and 0.741 in the external validation cohort (**Supplementary Figures 5A**, **C**), respectively. At the same time, the discrimination between nomogram and other independent prognostic factor were compared, showing a better discriminative ability than the other independent factors at 3 and 5 years, both in the primary cohort (**Figures 3B**, **D**), the internal validation cohort (**Supplementary Figures 4B**, **D**) and the external validation cohort (**Supplementary Figures 5B**, **D**). These results demonstrated the good discrimination of nomogram. The calibration curves of primary cohort and validation cohort showed a good consistency between the predicted values of nomogram and the observed values (**Figure 4**). And the DCA curve displayed that the clinical prediction ability of our model was better, compared with AJCC stage and SEER summary stage (**Figure 5**).

### Risk Stratification by Nomogram

**Figure 6:**
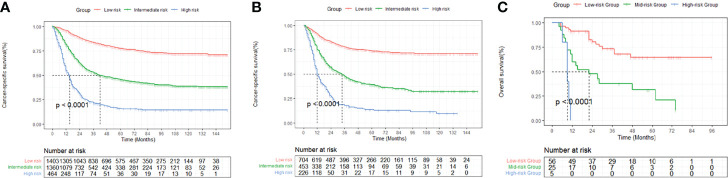
Kaplan-Meier curves of cancer-specific survival for patients in the low-, intermediate-, and high-risk groups in the primary cohort **(A)**, the internal validation cohort **(B)** and the external validation cohort **(C)**.

In order to improve the management of postoperative patients with bladder cancer, we developed a risk stratification system based on the points of nomogram. The best cut-off value was determined by X-tile and patients were divided into three groups: low-risk group (total scores<147), intermediate-risk group (147≤total scores ≤ 225), high-risk group (total scores>225) (**Supplementary Figure 6**). The Kaplan-Meier curves displayed that whether in the training set or the validation set, the prognosis of the three risk groups was significantly different (P < 0.0001, **Figure 6**).

### Online Application of Nomogram

To make it easier to apply our nomogram, we established a web calculator which can be accessed at https://chentao.shinyapps.io/BLCA_DynNomapp/. We can obtain the corresponding survival probability by just entering the available raw data.

## Discussion

Because of the high incidence rate of BLCA, the risk stratification of bladder urothelial carcinoma after RC has essential clinical significance (19). It is conducive to follow-up consultation, postoperative treatment and clinical trials. For BLCA patients receiving RC, the prognosis not only correlates with T stage and biological characteristics (14, 15), but also with the degree of lymph node dissection (5, 11). However, so far, effective and objective indicators to evaluate the impact of individualized lymph node resection are still lacking.

In this study, we defined LONT as the log of the ratio between the NLN counts plus one and the T stage, where NLNs represents the degree of LND and T stage represents tumor characteristics and the disease severity of tumor (16). Compared with the simple application of ELNs (11) and LODDS (12) to represent the degree of LND, LONT took into account the characteristics of individualized tumor. It can be understood as the NLNs adjusted by T stage, representing the relative number of negative lymph nodes removed for postoperative BLCA patients. A higher value of LONT indicates a larger relative number of negative lymph nodes, and a lower value indicates a smaller relative number of negative lymph nodes. The same LONT value may represent the same degree of risk even if the patient’s T stage and the number of negative lymph nodes are different, which was conducive to comparing the individualized degree of LND in patients with different TNM stages. In addition, we further established a simple and effective prognostic model based on LONT. The model was composed of nine prognostic factors, including LONT, age, race, tumor size, histologic.type, T.stage, N.stage, summary.stage and chemotherapy, which were obtained based on univariate and multivariate Cox regression analysis. We also compared our model with the AJCC stage and SEER summary stage on clinical performance, the results of C-index, AUC and DCA proved that our nomogram had a better prediction value. Moreover, we further constructed a risk stratification system to divide patients into low-, middle- and high-risk groups, which was instrumental in the choice of patients’ treatment.

To our knowledge, only one study had explored the role of LONT in the prognosis of postoperative patients with gastric cancer (16). Similar to its research, we found that this new indicator was an independent prognostic factor in postoperative BLCA patients. The result of univariate Cox regression analysis suggested that the HR of NLNs in OS and CSS was 0.983 and 0.984, respectively. Interestingly, when we combined T stage with NLNs, its HR decreased significantly to 0.354 and 0.339. Similar results also occurred in the multivariate Cox regression and validation cohort. At the same time, the prognosis of BLCA patients after RC could be significantly stratified by LONT both in CSS and OS cohorts. The proportion of LONT in our nomogram was also largest. In addition, this prognostic factor had strong clinical availability and can be obtained through a simple calculation of postoperative pathology. All these results indicated the importance of LONT in predicting postoperative bladder cancer, which can further improve the accuracy of prediction.

In recent years, many researchers have been devoted to establishing a predictive model for postoperative bladder cancer (19–23). Although these models had been well validated externally, they may not be universally applicable. Because some included variables were usually unavailable, such as adjuvant radiotherapy and lymphatic vascular invasion, and the calculation is relatively complicated (24). In addition, Yang et al. constructed a prognostic model to assess the cancer-specific survival for BLCA patients after RC (24). The final model consisted of five variables including T stage, marital status, N stage, tumor size and chemotherapy. The C index of our model is significantly higher than them, whether in the primary cohort (0.734 vs 0.718) or validation cohort (0.720 vs 0.707). Compared with the above models, our study may have the following advantages. Firstly, we were the first to introduce a novel and significant factor (LONT) to predict CSS in BLCA patients receiving RC. This variable took full account of the individual LND effect. Secondly, we further developed a risk stratification system according to the nomogram score, which may help clinicians identify high-risk groups in time.

Indeed, some shortcomings should be acknowledged in our study. Firstly, it may be inaccurate for us to only represent the characteristics of tumor by T stage, because the known important biological characteristics of tumor also include pathological type, grade, genotyping, etc. Secondly, some potentially important prognostic factors were not included in our study, such as complications and lymphatic vascular invasion. In addition, the SEER database lacks specific information about surgery and chemotherapy. The sequence of chemotherapy and the choice of surgical methods and chemotherapy drugs may have different effects on the prognosis (25–27). Lastly, even if we carried out external validation, further prospective studies were required to verify our conclusions.

## Conclusion

As a novel prognostic indicator, LONT can fully reflect the degree of individualized lymph node dissection in different patients. Based on LONT, a more effective nomogram was developed and validated to predict 3- and 5- CSS probabilities, which is conducive for clinicians to formulate individualized treatment plans.

## Data Availability Statement

The original contributions presented in the study are included in the article/**Supplementary Material**. Further inquiries can be directed to the corresponding authors.

## Ethics Statement

This study was approved by the institutional of the First Affiliated Hospital of Nanchang University. All data from SEER was open access.

## Author Contributions

TC and XZ conceived the study. HW and TC collected and analyzed the data. XZ and MJ drafted the manuscript. TC and LC generated the tables and figures. BF supervised the study. All authors approved the final version. All authors contributed to the article and approved the submitted version.

## Funding

This study was supported by grants from the National Natural Science Foundation of P.R. China (Grant Nos.81560419).

## Conflict of Interest

The authors declare that the research was conducted in the absence of any commercial or financial relationships that could be construed as a potential conflict of interest.

## Publisher’s Note

All claims expressed in this article are solely those of the authors and do not necessarily represent those of their affiliated organizations, or those of the publisher, the editors and the reviewers. Any product that may be evaluated in this article, or claim that may be made by its manufacturer, is not guaranteed or endorsed by the publisher.

## Supplementary Material

Supplementary Figure 1 Patients selection flowchart.

Supplementary Figure 2 The optimal cutoff values of age and tumor size were estimated by Kaplan-Meier curves utilizing X-tile software. **(A, B)** The optimal cut-off values of age were 63 and 76 years old. **(C, D)** The optimal cut-off values of tumor size were 30 and 64mm.

Supplementary Figure 3 The optimal cutoff values of LONT were estimated by Kaplan-Meier curves utilizing X-tile software.

Supplementary Figure 4 Time-dependent ROC curves of the nomogram predicting 3-years **(A)**, and 5-years **(C)** CSS in the internal validation cohort. Comparison of the ROC curves between nomogram and other independent factors at the 3-years **(B)**, and 5-years **(D)** CSS in the internal validation cohort.

Supplementary Figure 5 Time-dependent ROC curves of the nomogram predicting 3-years **(A)**, and 5-years **(C)** CSS in the external validation cohort. Comparison of the ROC curves between nomogram and other independent factors at the 3-years **(B)**, and 5-years **(D)** CSS in the external validation cohort

Supplementary Figure 6 The optimal cutoff values of the score calculated by the nomogram were estimated by Kaplan-Meier curves utilizing X-tile software.

## Abbreviations

BLCA, Bladder cancer; CSS, Cancer-specific survival; CSM, Cancer-specific mortality; OS, Overall survival; ELNs, Examined lymph nodes; NLNs, Negative lymph nodes; LONT, Log odds of negative lymph nodes/T stage; RC, Radical cystectomy; LND, Lymph node dissection; ePLND, extended pelvic lymph node dissection; IOR, Interquartitle range; CI, Confidence interval; OR, Odds ratio; Other^+^, American/Indian/Alaska/Native/Asian/Pacific Islands; SDW, separated, divorced or widowed; TCC, Transitional cell carcinoma; PTCC, Papillary transitional cell carcinoma.
