# Supplementary figures and images for: Predictive Value of the Log Odds of Negative Lymph Nodes/T Stage as a Novel Prognostic Factor in Bladder Cancer Patients After Radical Cystectomy
Source: Front Oncol. 2022 Jul 19;12:895413. doi: 10.3389/fonc.2022.895413 (PMC9343753; doi:10.3389/fonc.2022.895413)

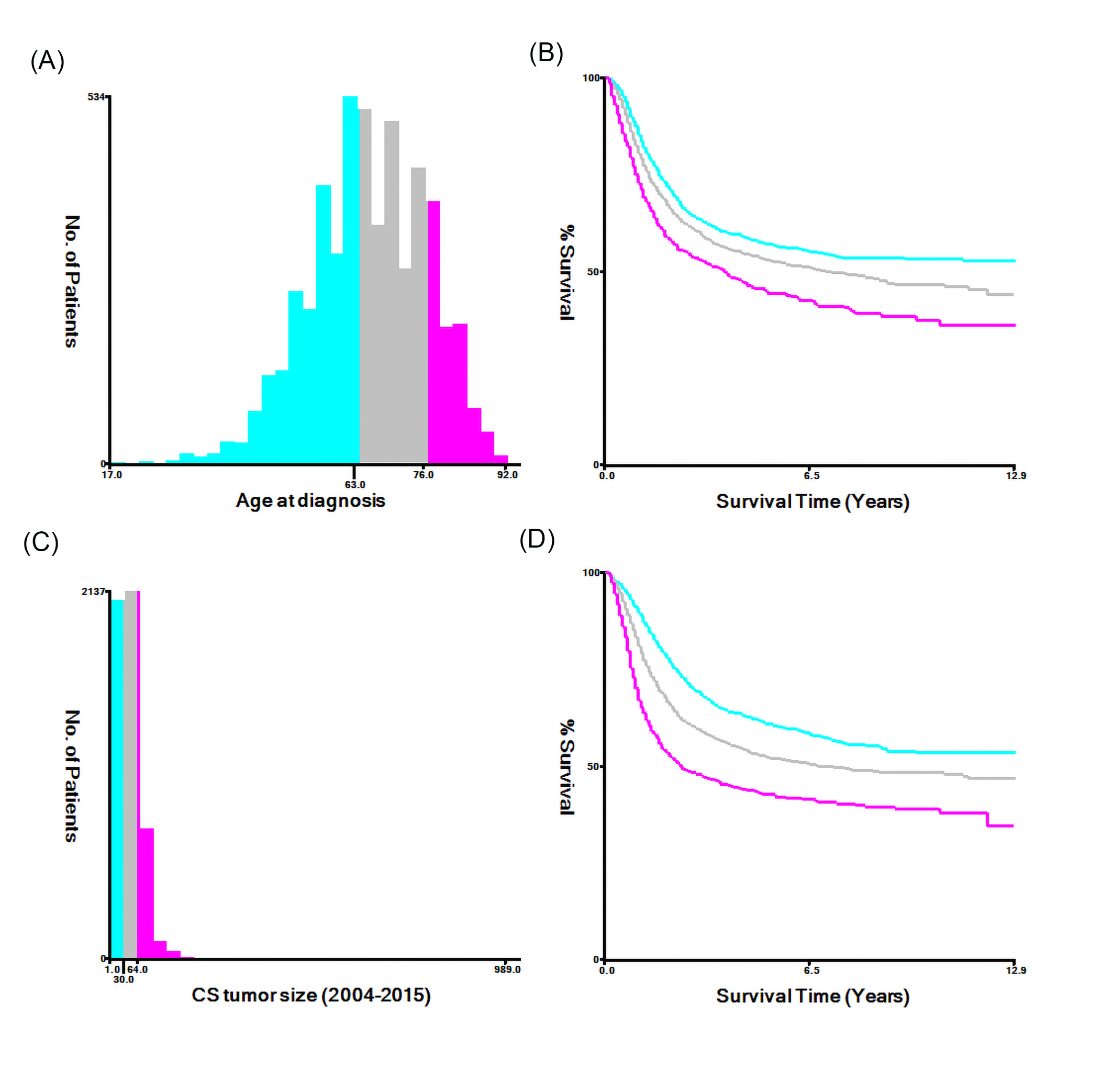

Supplement: Supplementary Figure 2 — The optimal cutoff values of age and tumor size were estimated by Kaplan-Meier curves utilizing X-tile software. (A, B) The optimal cut-off values of age were 63 and 76 years old. (C, D) The optimal cut-off values of tumor size were 30 and 64mm. [file Image_2.tif]

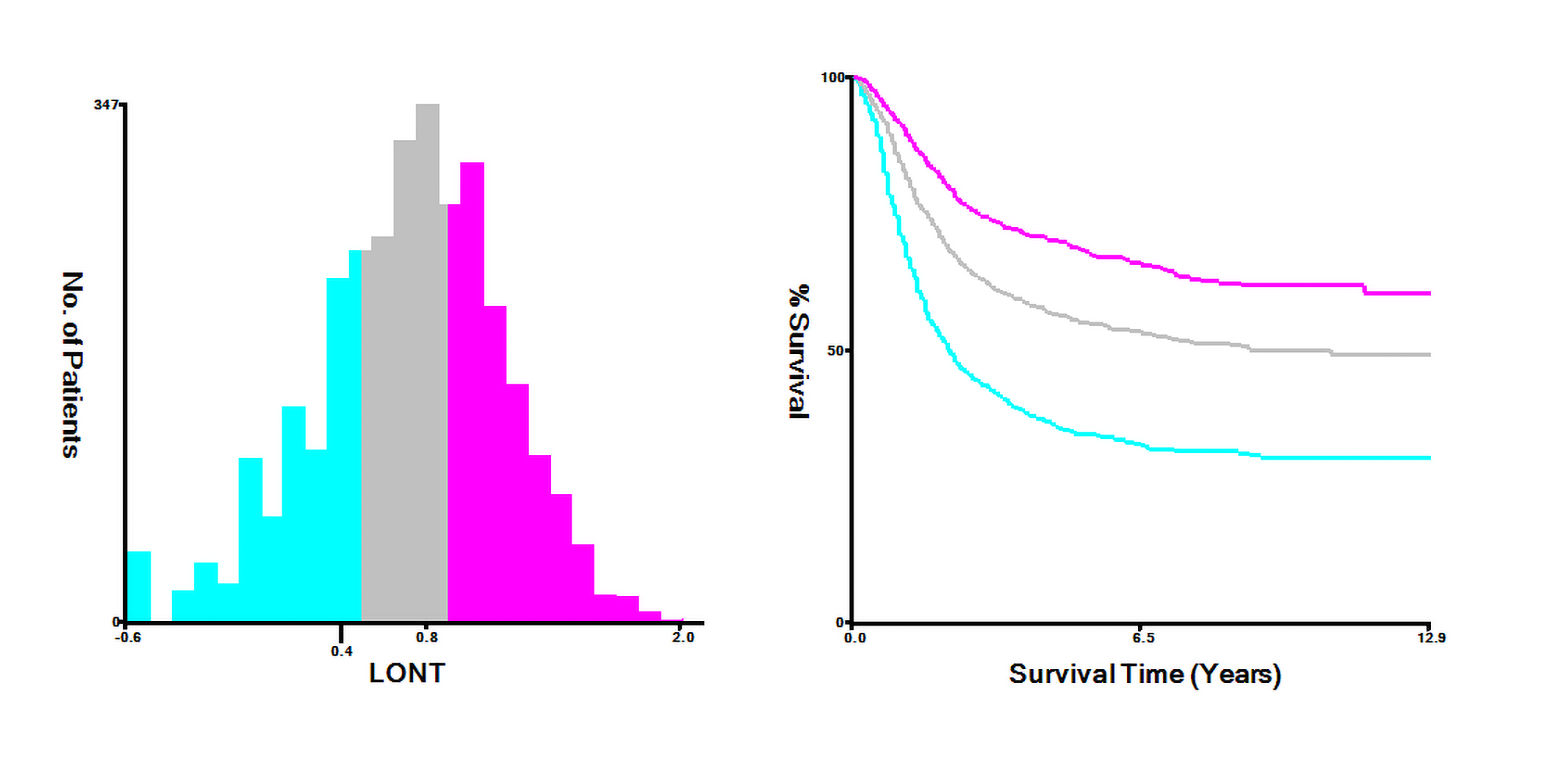

Supplement: Supplementary Figure 3 — The optimal cutoff values of LONT were estimated by Kaplan-Meier curves utilizing X-tile software. [file Image_3.tif]

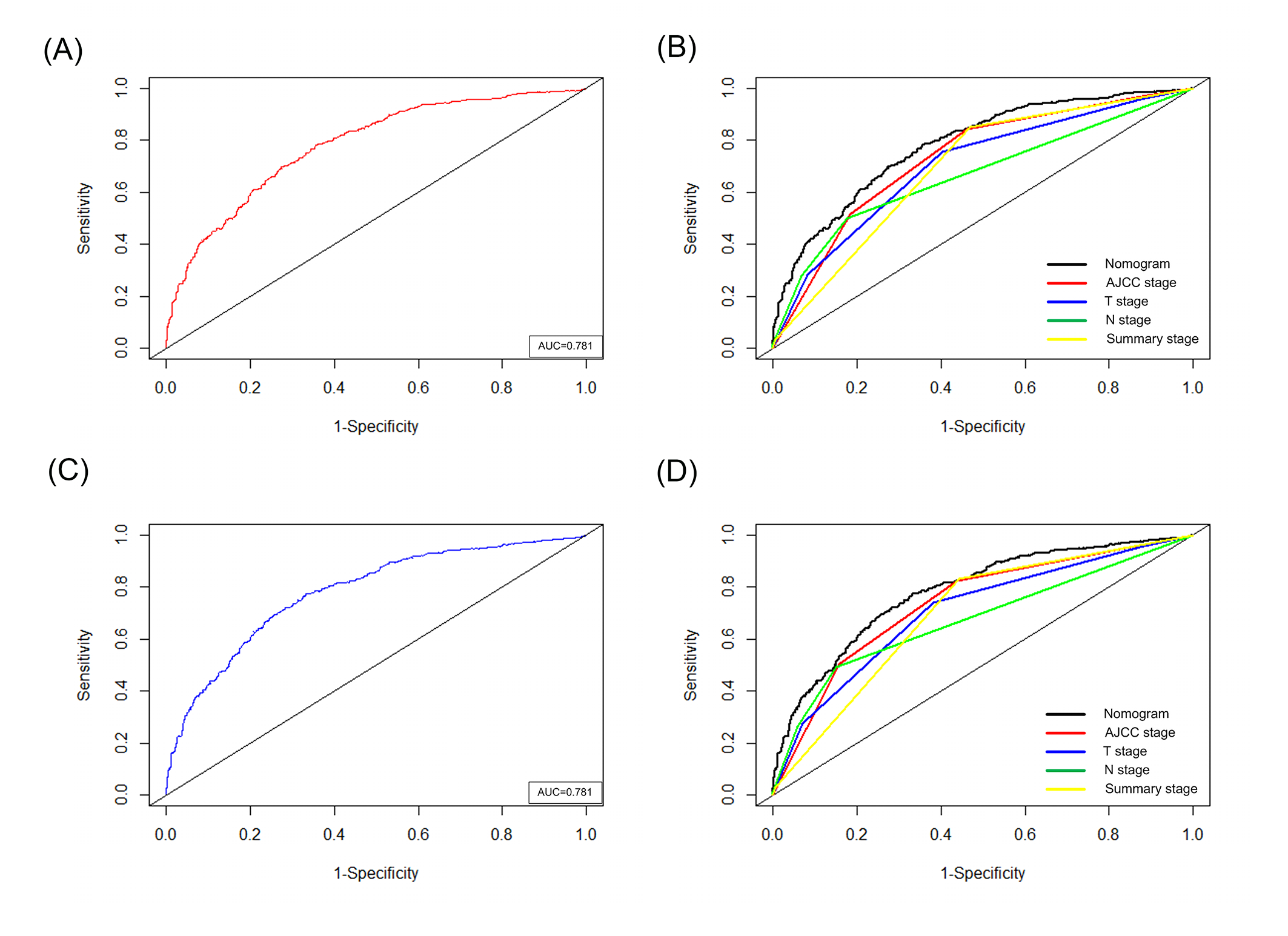

Supplement: Supplementary Figure 4 — Time-dependent ROC curves of the nomogram predicting 3-years (A), and 5-years (C) CSS in the internal validation cohort. Comparison of the ROC curves between nomogram and other independent factors at the 3-years (B), and 5-years (D) CSS in the internal validation cohort. [file Image_4.tif]

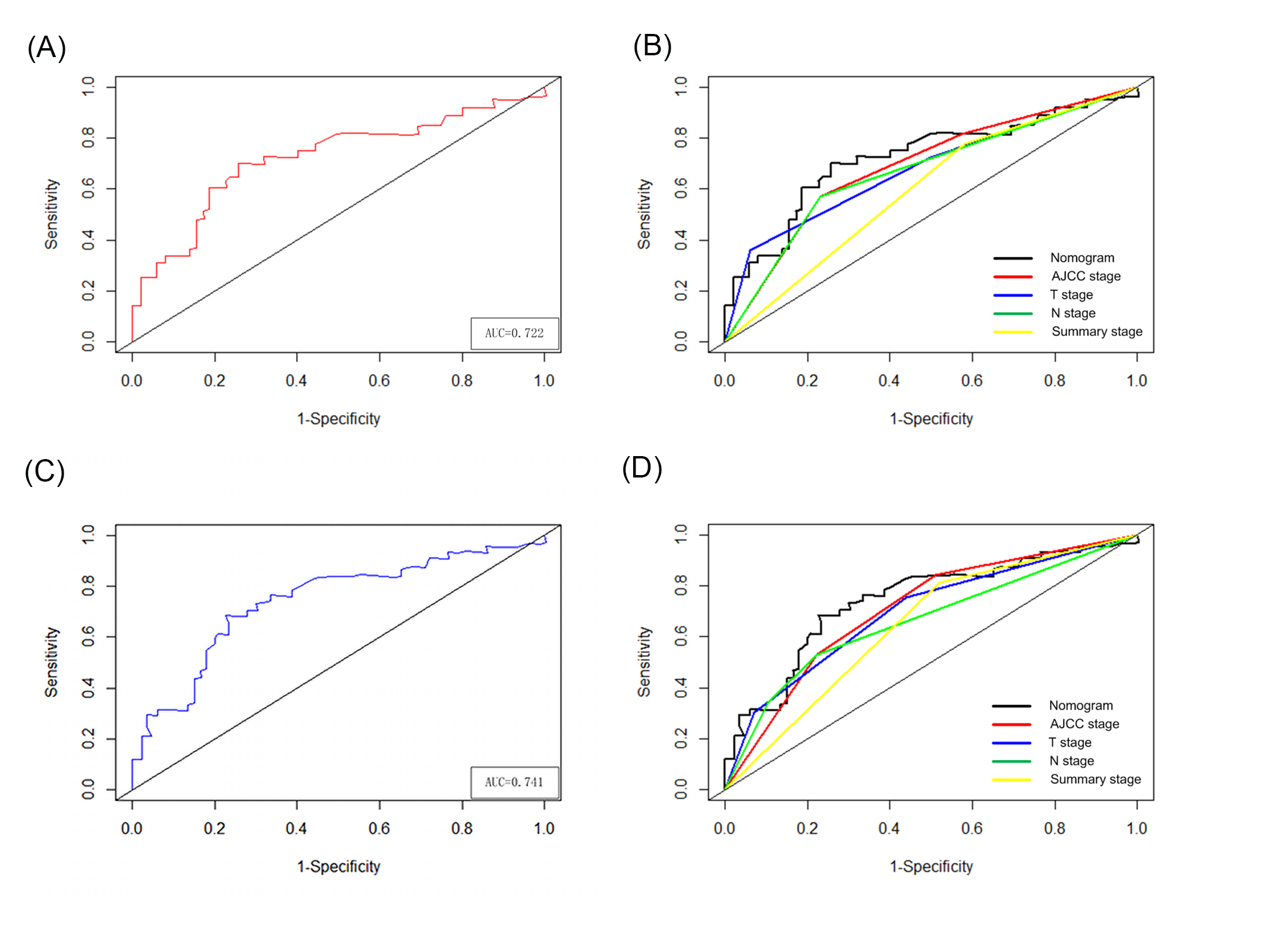

Supplement: Supplementary Figure 5 — Time-dependent ROC curves of the nomogram predicting 3-years (A), and 5-years (C) CSS in the external validation cohort. Comparison of the ROC curves between nomogram and other independent factors at the 3-years (B), and 5-years (D) CSS in the external validation cohort [file Image_5.tif]

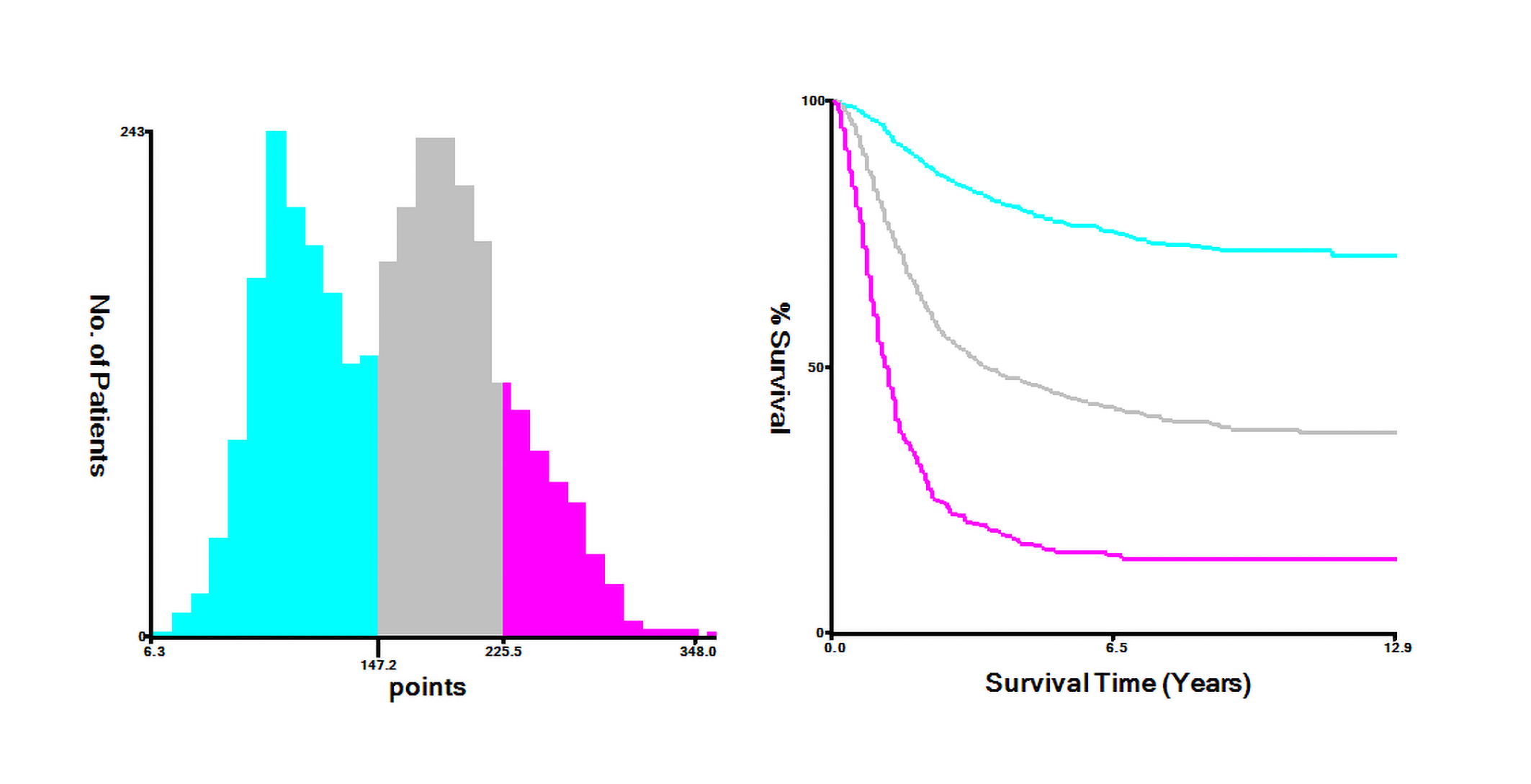

Supplement: Supplementary Figure 6 — The optimal cutoff values of the score calculated by the nomogram were estimated by Kaplan-Meier curves utilizing X-tile software. [file Image_6.tif]
